# Continuing early mTICI 2b recanalization may improve functional outcome but is associated with a higher risk of intracranial hemorrhage

**DOI:** 10.3389/fneur.2022.955242

**Published:** 2022-09-26

**Authors:** Paul Steffen, Noel Van Horn, Rosalie McDonough, Milani Deb-Chatterji, Anna Christina Alegiani, Götz Thomalla, Jens Fiehler, Fabian Flottmann

**Affiliations:** ^1^Department for Diagnostic and Interventional Neuroradiology, University Medical Center Hamburg-Eppendorf, Hamburg, Germany; ^2^Foothills Medical Centre, Alberta Health Services, Calgary, AB, Canada

**Keywords:** mechanical thrombectomy, acute ischemic stroke, modified Thrombolysis in Cerebral Infarction (mTICI), digital subtraction angiography, early mTICI 2b

## Abstract

**Background:**

Successful reperfusion (mTICI 2c/3) and low number of passes are key determinants for good clinical outcome in acute large vessel occlusion. While final mTICI 2c/3 reperfusion is superior to partial reperfusion (mTICI 2b) it remains unclear if this is also true for the subgroup of patients with early mTICI 2b (achieved in ≤2 retrieval attempts) reperfusion who are secondarily improved to mTICI 2c/3. This study was designed to examine if early mTICI2b should be continued or stopped during mechanical thrombectomy (MT).

**Methods:**

Nine hundred and thirteen ischemic stroke patients who received MT were retrospectively analyzed. Angiography runs following each recanalization attempt were scored for mTICI. The patients with early mTICI 2b reperfusions were dichotomized in “TICI2b-stopped” (MT withdrawal after mTICI 2b was achieved with first or second retrieval) and “TICI2b-continued” (MT was continued after mTICI 2b was achieved with first or second retrieval). Functional outcome was obtained after 90 days using the modified Rankin scale (mRS90).

**Results:**

Of 362 Patients with a M1-occlusion, 100 patients fulfilled the inclusion criteria with an early mTICI 2b. 78/100 patients were included in the “TICI2b-stopped” group and 22/100 patients were in the “TICI2b-continued” group. Of these 22 patients, none had a final mTICI score lower than 2b and 11 patients had a final mTICI score of 2c/3. Regarding good functional outcome at mRS90, “TICI2b-continued” showed by trend a slight advantage of 40.1 vs. 35.6% in “TICI2b-stopped” but in multivariate logistic regression analysis adjusted for confounders, no significant difference was found between the two groups (OR 0.75, 95% CI 0.19–2.87, *p* = 0.67). Symptomatic intracranial hemorrhage was significantly higher in “TICI2b-continued” compared to “TICI2b-stopped” (31.8 vs. 10.3%, *p* = 0.031).

**Conclusion:**

Successfully improving an early mTICI 2b to mTICI 2c/3 reperfusion is possible in a substantial number of patients and might improve functional outcome. However, an increase in symptomatic intracranial hemorrhage (SICH) due to further retrieval attempts may diminish the potential functional benefit to continue early mTICI 2b. To support this finding, further investigation with more power is needed to account for the low number of events regarding SICH.

## Introduction

Recently, randomized controlled trials have established mechanical thrombectomy (MT) as standard of care in acute ischemic stroke patients with large vessel occlusion (1) and successful recanalization is considered the most important determinant of functional outcome (2). An established imaging tool to evaluate reperfusion and predict functional outcome during MT is the modified Thrombolysis in Cerebral Infarction (mTICI) score (3), which ranges from 0 (no reperfusion) to 3 (complete reperfusion); functional outcome improves with higher mTICI scores and a mTICI score of 2c/3 is often referred to as “successful reperfusion” (4, 5). However, often several attempts are needed for successful reperfusion (6) and the number of retrieval attempts correlate negatively with functional outcome (7). This is most likely due to an increase in complications such as intracranial hemorrhage and thrombus fragmentation (8, 9).

In clinical practice, it is often difficult to determine when to abort or continue MT, especially when partial reperfusion (mTICI 2b) is achieved. The interventionalist must weigh the potential benefits of a final mTICI 2c/3 reperfusion over a final mTICI 2b against the possible detrimental effects of further retrieval attempts (10, 11). The success rate of mTICI 2b to mTICI 2c/3 conversion, as well as its associated effect on clinical outcome and complications are unknown. This retrospective study analyzes reperfusion grades after each retrieval attempt and its associated functional outcome, to provide guidance for neurointerventionalists regarding the clinical question: Is it better to continue or to stop after early mTICI 2b reperfusion was achieved in MT? We hypothesized that MT should be continued after early mTICI 2b (mTICI 2b with first or second retrieval) was achieved to improve functional outcome.

## Methods

### Study population

We retrospectively analyzed institutional data of all 913 consecutive patients referred to our hospital between December 2015 and September 2020 who received endovascular treatment of acute large vessel occlusion via stent retriever and/or aspiration thrombectomy. Data was obtained as part of the German Stroke Registry - Endovascular Treatment (Ethics committee LMU Munich, approval number 689-15). We included all patients with an isolated middle cerebral artery occlusion who had mTICI 2b reperfusion after the first or second retrieval attempt. Patients with occlusions of additional vascular territories and recurrent stroke were excluded.

Baseline demographic data, National Institutes of Health Stroke Scale (NIHSS), Alberta Stroke Program Early CT Score (ASPECTS), administration of intravenous thrombolysis, and modified Ranking Scale (mRS) were documented during the clinical routine. Good clinical outcome was defined as mRS of ≤ 2 at 90 days (mRS90) after stroke onset and was assessed as the primary outcome. Intracranial hemorrhage was classified according to the Heidelberg Bleeding Classification and was assessed 24 h after MT (12). Symptomatic intracranial hemorrhage (SICH) was defined as new intracranial hemorrhage detected on follow-up CT scan as well as an increase in NIHSS of at least four points.

### Image analysis

Two observers (F.F. and P.S.) with 8 and 5 years of stroke imaging experience, independently evaluated all pre-interventional stroke images of non-enhanced computed tomography (CT) scans for ASPECTS as well as all digital subtraction angiographies performed during MT. Reperfusion results were classified after each retrieval attempt according to the mTICI score ranging from 0 (no reperfusion) to 3 (complete reperfusion), as recently proposed (3, 13, 14). A reperfusion result of mTICI 2 was therefore divided in 2a (<50% reperfusion), 2b (>50% reperfusion), and 2c (near complete reperfusion except slow flow or few distal cortical emboli) (14). Discrepancies between raters were resolved by consensus. Patients with mTICI 2b after ≤2 retrieval attempts were included for further analysis and dichotomized into “TICI2b-stopped” (MT was aborted after mTICI2b was achieved) and “TICI2b-continued” (MT was continued after mTICI 2b was achieved to further improve reperfusion). All digital subtraction angiography runs were rated for complications such as vasospasm, dissection, or perforation.

Malignant swelling and intracranial hemorrhage according to Heidelberg Bleeding Classification were reported on follow-up CTs after 24 h (15). The Classification discriminates between hemorrhagic transformation (H1 = scattered small petechiae without mass effect, H2 = confluent petechiae without mass effect), hematoma within infarcted tissues (PH1 = hematoma occupying <30% of infarct volume without substantial mass effect, PH2 = hematoma occupying more than 30% of infarcted area with obvious mass effect), and hematoma outside of the infarcted brain tissue (3A = parenchymal hematoma, 3B = intraventricular hemorrhage, 3C = subarachnoid hemorrhage, 3D = subdural hemorrhage) (12).

### Statistical analysis

All analyses were performed with the R statistics program (v.3.6.3, R Core Team 2019, Vienna Austria; RStudio IDE v. 1.1.463, Boston, MA, USA). Normally distributed variables are reported as mean and standard deviation (SD) and compared using the Student's *t*-test. Non-normally distributed data are reported as median and interquartile range (IQR) and compared using Wilcoxon rank sum test. Categorical variables are reported as proportions and compared using Fisher's Exact Test. Association between clinical/radiological parameters and outcome was assessed using a multivariable logistic regression analysis with good functional outcome at 3 months (mRS90) as the dependent variable. The variables for the multivariate analysis as predictor variables included age, sex, NIHSS on admission, mRS before admission, arterial hypertension, diabetes, dyslipidemia, atrial fibrillation, intravenous thrombolysis, and ASPECTS. *P*-values <0.05 were considered significant.

## Results

### Group characteristics

**Figure 1:**
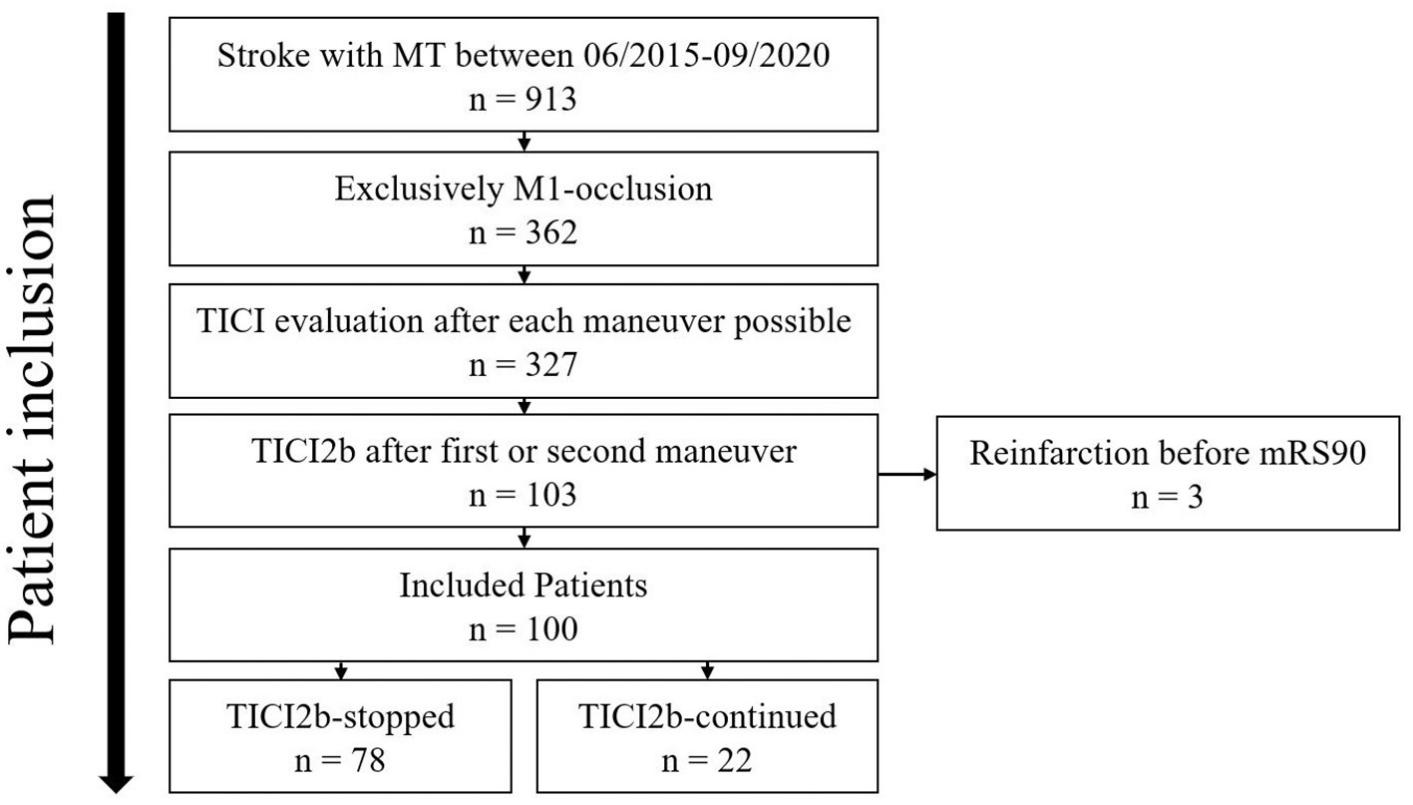
Patient selection. MT, mechanical thrombectomy; TICI, Thrombolysis in Cerebral Infarction; mRS90, modified Ranking Scale at day 90. Of 913 consecutive patients who received MT, 100 patients met the inclusion criteria and were distributed into TICI2b-stopped and TICI2b-continued.

Of 913 patients analyzed, 100 met inclusion criteria. Of these, 78 patients (78%) were included in the “TICI2b-stopped” and 22 patients (22%) in the “TICI2b-continued” group. Patient selection and exclusion are illustrated in Figure 1.

Baseline patient data and group characteristics are displayed in Table 1. Significant differences between the two groups were only observed for ASPECTS (TICI2b-stopped: 8 vs. TICI2b-continued: 8.5, *p* = 0.037) and NIHSS (TICI2b-stopped: 15 vs. TICI2b-continued: 12, *p* = 0.015) on admission. Overall group characteristics were similar to the recently published nationwide database of patients who received MT (16).

**Table 1:** Baseline patient data, stratified according to TICI2b-stopped and TICI2b-continued.

	**ALL**	**TICI2b-stopped**	**TICI2b-continued**	***p*-value**
	***N =* 100**	***N =* 78**	***N =* 22**	
Age, mean y (±SD)	73 (13)	74 (13)	71 (15)	0.397
Female sex, *n* (%)	61 (61%)	50 (64%)	11 (50%)	0.342
**Comorbidities**, ***n*** **(%)**				
Arterial hypertension	64 (64%)	51 (65%)	13 (59%)	0.771
Diabetes	18 (18%)	16 (21%)	2 (9%)	0.347
Dyslipidaemia	13 (13%)	11 (14%)	2 (9%)	0.727
Atrial fibrillation	42 (42%)	36 (46%)	6 (27%)	0.180
Smoker (current)	10 (10%)	8 (10%)	2 (9%)	0.866
Admission ASPECTS, median (IQR)	8 (7; 9)	8 (6; 9)	9 (8; 9)	0.037*
Admission NIHSS, median (IQR)	14 (11; 17)	15 (11; 18)	12 (8; 15)	0.015*
IVT, *n* (%)	54 (54%)	41 (53%)	13 (59%)	0.764

TICI, Thrombolysis in Cerebral Infarction; ASPECTS, Alberta Stroke Program Early CT Score; IQR, interquartile range; NIHSS, National Institutes of Health Stroke Scale; IVT, intravenous thrombolysis. Data are displayed as mean (± standard deviation) for age and as median (IQR) for ASPECTS and NIHSS. Sex, comorbidities and IVT are displayed as n, frequencies (%).

* Significant difference between groups (*p* < 0.05).

### Procedural outcome

**Figure 2:**
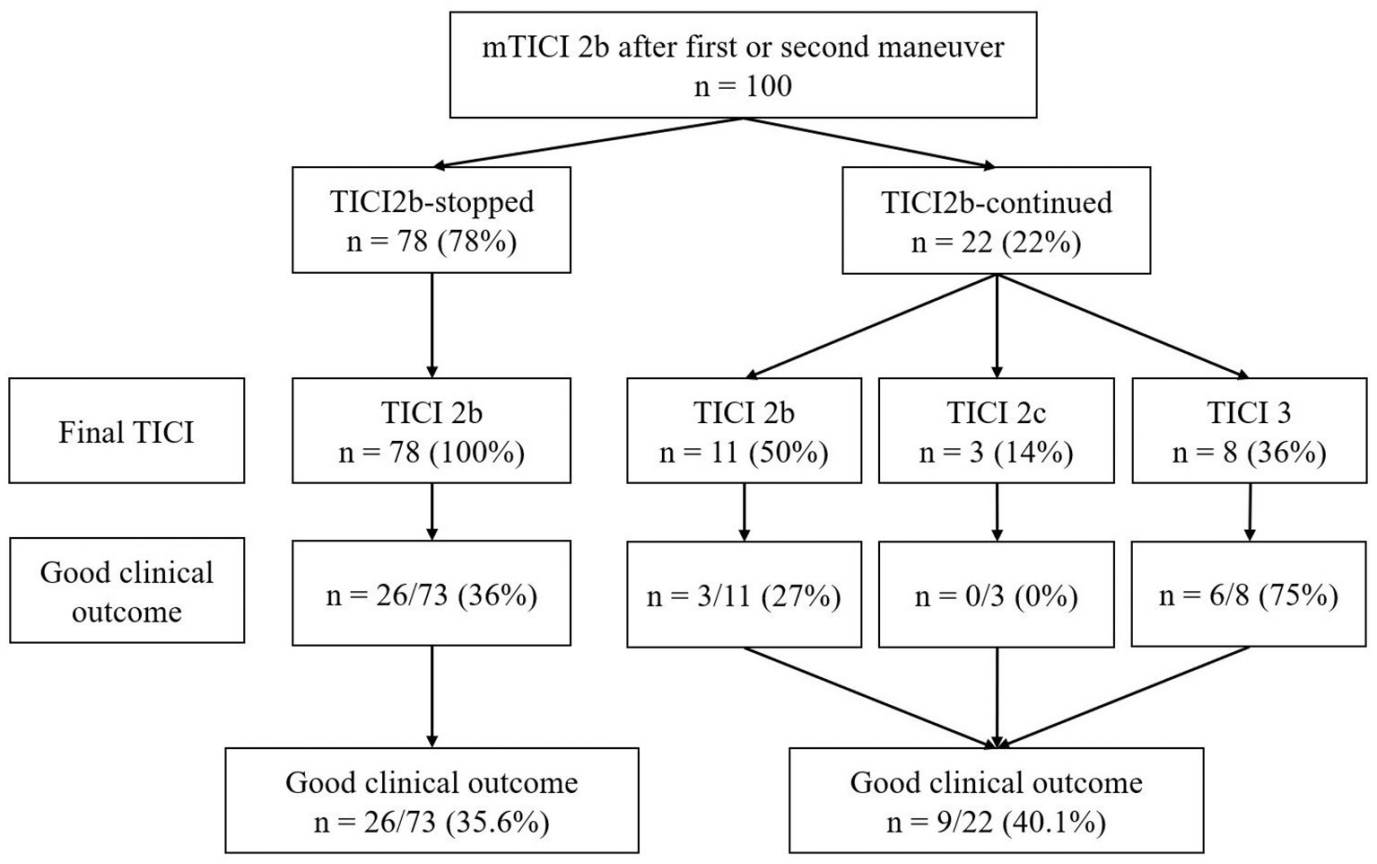
Final mTICI score and function outcome. TICI, Thrombolysis in Cerebral Infarction. Final mTICI score and functional outcome stratified for “TICI2b-stopped” and “TICI2b-continued”. In “TICI2b-continued” final mTICI was never below mTICI 2b and an improvement to mTICI 2c/mTICI 3 was achieved in 50% of patients. Good functional outcome 90 days after stroke onset (mRS90 ≤ 2) was not significantly different between groups (*p* = 1.000).

Final mTICI scores and functional outcomes are illustrated in Figure 2. The final reperfusion result for “TICI2b-stopped” was always mTICI 2b, as defined by study protocol. In the “TICI2b-continued” group, final mTICI was 2b in 11 patients (50.0%), 2c in three patients (13.6%), and 3 in eight patients (36.4%). Final mTICI score was significantly different between “TICI2b-stopped” and “TICI 2b-continued” (*p* < 0.001).

### Functional outcome

**Figure 3:**
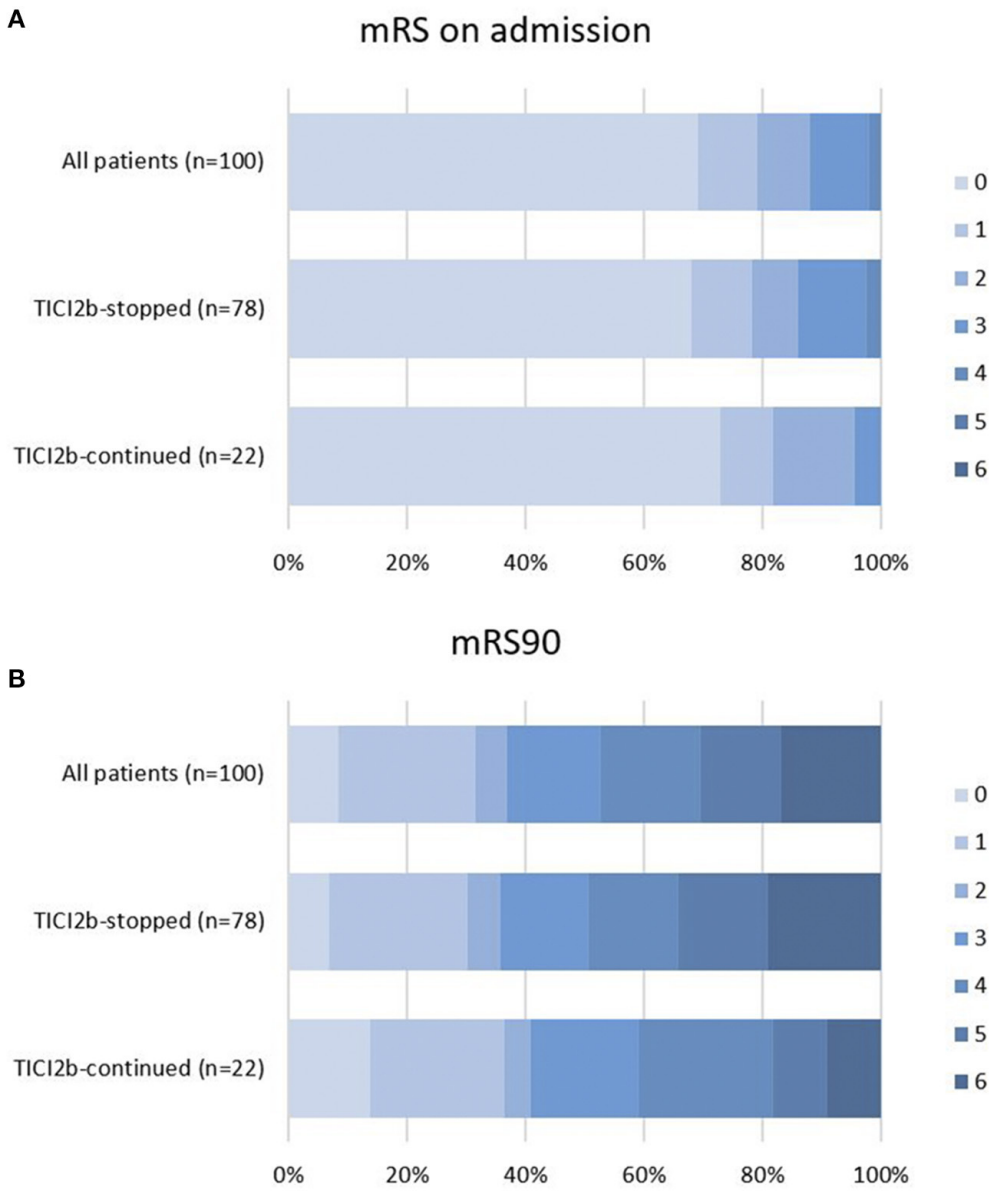
Functional outcome. Functional outcome according to mRS in percentage of patients on admission **(A)** and 90 days after stroke onset **(B)** stratified according to “TICI2b-stopped” and “TICI2b-continued” mRS90, modified Ranking Scale.

Good functional outcome was observed in 35.6% in the “TICI2b-stopped” and 40.1% in the “TICI2b-continued” group, with no significant difference between the two (*p* = 1.000). Good functional outcome increased with increasing final mTICI grades from 34.5% (mTICI 2b) to 75% (mTICI 3). All three patients with a final mTICI score of 2c had a mRS90 of 3. The median mRS90 was 3 for “TICI2b-stopped” (IQR 1;5) and 3 for “TICI2b-continued” (IQR 1;4), respectively (*p* = 0.257). The mortality rate at day 90 was 9.1% (2/22) in the “TICI2b-continued” group and 19.2% (14/73) in the “TICI2b-stopped” group (*p* = 0.433). The exact distribution of mRS before admission and after 90 days are illustrated in Figures 3A,B.

Results of multivariable logistic regression analysis are depicted in Table 2. Good clinical outcome (mRS 0–2) was assossiated with NIHSS on admission (OR 0.76, 95% CI 0.65–0.87; *p* = 0.000) and mRS before admission (OR 0.24, 95% CI 0.05–0.64; *p* = 0.022). No significant difference was found between TICI2b-continued and TICI2b-stopped (OR 0.75, 95% CI 0.19–2.87; *p* = 0.668).

**Table 2:** Logistic regression analysis with odds ratio and *p*-value for analyzed data.

**Variables**	**OR**	**CI**		***p*-value**
		2.5%	97.5%	
Age, years	0.95	0.90	1.00	0.052
Sex, male	0.98	0.27	3.42	0.980
NIHSS on Admission	0.76	0.65	0.87	0.000*
mRS before Admission	0.24	0.05	0.64	0.022*
Arterial Hypertension	2.63	0.68	11.20	0.171
Diabetes	0.35	0.05	1.79	0.227
Dyslipidemia	1.30	0.20	8.85	0.785
Atrial fibrillation	1.64	0.45	6.30	0.454
IVT	1.81	0.51	6.79	0.364
ASPECTS	1.28	0.85	2.00	0.253
TICI2b-continued	0.75	0.19	2.87	0.667

Calculated OR, CI 2.5%, CI 97.5%, and p-value of all analyzed variables via multivariable regression analysis. OR, Odds Ratio; CI, confidence interval; NIHSS, National Institutes of Health Stroke Scale; mRS, modified Rankin Scale; IVT, intravenous thrombolysis; ASPECTS, Alberta Stroke Programme Early CT Score.

* Significant difference between groups (*p* < 0.05).

### Number of retrievals and complications

The median number of retrieval attempts was 1 (IQR 1;1) for “TICI2b-stopped” and 3 (IQR 2;3) for “TICI2b-continued” (*p* < 0.001). During the procedure, adverse events were described in 4 (4%) patients. Periprocedural complications were vasospasm, perforation, dissection, and intracranial hemorrhage. Three patients showed various combinations of these complications. Symptomatic intracranial hemorrhage was significantly higher in “TICI2b-continued” compared to “TICI2b-stopped” (32 vs. 10%, *p* = 0.031). By trend, PH1, PH2, and 3B (intraventricular) type bleeds were higher in the “TICI2b-continued” group compared to the “TICI2b-stopped” group. All three patients with PH2 hemorrhage showed signs of 3B and/or 3C hemorrhage. Type 3C (subarachnoid) hemorrhage was seen in 6% of patients in the “TICI2b-stopped” and in 32% in the “TICI2b-continued” group, showing a significant difference between groups (*p* = 0.004). The overall incidence of Type 3C bleeding in the cohort was 12%. Malignant infarction was seen in one patient of “TICI2b-stopped” and two patients of “TICI2b-continued.” Exact distribution of complications and retrieval attempts are displayed in Table 3.

**Table 3:** Retrieval attempts and complications.

	**ALL**	**TICI2b-stopped**	**TICI2b-continued**	***p*-value**
	***N* = 100**	***N* = 78**	***N* = 22**	
Retrieval attempts, median (IQR)	1 (1; 2)	1 (1; 1)	3 (2; 3)	<0.001
**Periprocedural complications**				
vasospasm, *n* (%)	2 (2%)	2 (3%)	1 (5%)	1.000
perforation, *n* (%)	3 (3%)	1 (1%)	2 (9%)	0.235
dissection, *n* (%)	1 (1%)	1 (1%)	0	1.000
intracranial hemorrhage, *n* (%)	3 (3%)	1 (1%)	2 (9%)	0.235
**ICB and complications after 24 h**				
H1, *n* (%)	11 (11%)	9 (12%)	2 (9%)	1.000
H2, *n* (%)	5 (5%)	5 (6%)	0 (0%)	0.506
PH1, *n* (%)	8 (8%)	7 (9%)	1 (5%)	0.817
PH2, *n* (%)	3 (3%)	1 (1%)	2 (9%)	0.235
3B, *n* (%)	3 (3%)	1 (1%)	2 (9%)	0.235
3C, *n* (%)	12 (12%)	5 (6%)	7 (32%)	0.004
SICH, *n* (%)	15 (15%)	8 (10%)	7 (32%)	0.031
Malignant middle cerebral artery infarction, *n* (%)	3 (3%)	1 (1%)	2 (9%)	0.235

TICI, Thrombolysis in Cerebral Infarction; ICB, intracranial bleeding; SICH, symptomatic intracranial hemorrhage. Data are displayed as n, % except otherwise stated. ICB is described according to the Heidelberg Bleeding Classification.

## Discussion

During MT for acute large vessel occlusion stroke, the decision to continue or abort mTICI 2b reperfusion still involves many unknowns and is thus characterized by a high degree of uncertainty (10).

Several previous studies observed that a final mTICI score of 2c or 3 is superior to mTICI 2b with respect to clinical outcome (17–19) and thus should be strived for according to the guidelines of the European Stroke Organization/European Society of Minimally Invasive Neurological Therapy (20). First-pass reperfusion is the goal (21) since increasing retrieval attempts diminish returns on clinical benefit (7, 11).

Of the 100 patients with early mTICI 2b reperfusion, 22% were continued (“TICI2b-continued”) in an attempt to improve the final result, while 78% were stopped (“TICI2b-stopped”). In the “TICI2b-continued” group, the rate of successful conversion from mTICI 2b to mTICI 2c/3 was 50%. The patients successfully converted to mTICI 2c/3 had higher rates of good functional outcome compared to TICI2b-stopped (55 vs. 36%). A similar improvement between mTICI 2b and “secondarily achieved” mTICI 2c/3 was described by Kaesmacher et al., with rates of good functional outcome of 28.7% for mTICI 2b and 46.5% for secondarily improved mTICI 2c/3 reperfusion (4). In a recently published multicenter cohort study (*n* = 1,225), the rate of good functional outcome in successful recanalization was 49.4% after one and 42.1% after two retrieval attempts. Good functional outcome further decreased with increasing retrieval attempts (7). The HERMES meta-analysis also described comparable rates of good functional outcome (46%) for patients undergoing MT (1).

In the current study, once mTICI 2b reperfusion was achieved and continued, the final mTICI score was never below mTICI 2b, suggesting a chance to improve upon reperfusion grade without a concurrent risk of worsening the reperfusion status. Complications such as thrombus fragmentation, vasospasm, dissection, perforation, or intracranial hemorrhage are often a result from vascular damage caused by thrombectomy devices and may overshadow the clinical benefit of secondarily improved reperfusions (22). Further retrieval attempts resulted in a significant increase of 3C intracranial hemorrhage in the group of “TICI2b-continued” compared to “TICI2b-stopped” which is consistent with other studies demonstrating that more than three retrieval attempts are associated with a significant increase in SICH (8). A meta-analysis including studies such as ESCAPE (23), EXTEND (24), and REVASCAT (25) show an average incidence of SICH around 5.6% in cohorts where 58.7–88% of patients were treated successfully with thrombectomy (26). The incidence of SICH, especially in the “TICI2b-continued” group seemed high compared to these studies. Yet, while the other studies included all patients treated with thrombectomy, the current study focuses on a subpar cohort from a treatment prospection, missing the most successfully treated patients (first pass mTICI2c/3). The rates of PH1/PH2 hemorrhage were comparable to other publications (4, 8, 14).

While the successfully improved mTICI 2b to mTICI 2c/3 reperfusions showed by trend a better clinical outcome, the data did not show a significant difference in good functional outcome between the “TICI2b-stopped” and “TICI2b-continued” groups which may be explained by the small sample size in the “TICI2b-continued” group of 22 and its associated limited power as well as a significant increase in SICH in the “TICI2b-continued” group compared to the “TICI2b-stopped” group. Other complications, such as perforations, dissections, or worsening of the reperfusion grade by further retrieval attempts did not show significant differences between groups and seem to have less impact on the overall clinical outcome. Thus, the potential benefit of improved reperfusion with further retrieval attempts must be thoroughly weighed against the possible adverse effects of vascular damage from multiple retrieval attempts. Pre-interventional imaging with an evaluation of infarct core lesion and collaterals could help in the decision-making process when to continue or stop MT; for example, if good collaterals are present, the overall benefit of increasing reperfusion from mTICI 2b to mTICI 3 may be decreased (as it is presumed that they will reduce the likelihood of infarct growth) (10). Future investigation on the topic when to abort or continue a mechanical thrombectomy should also consider the recently presented data of the CHOICE-Study (27), which demonstrated a benefit for clinical outcome and SICH in patients who received intra-arterial thrombolysis after thrombectomy (mTICI2b to 3). These findings may give interventionalist another treatment tool for partial recanalized patients other than further retrieval attempts and may alter the decision-making process when to abort or continue a mechanical thrombectomy.

This is a retrospective observational, single-center study and as such has associated limitations. Firstly, mTICI 2b reperfusions are heterogenous, a topic of ongoing investigation we attempted to partially address by using the modified (m)TICI score for this study. Secondly, the sample size, especially in the “TICI2b-continued” group was small, limiting the power of the study to detect significant differences in clinical outcome. Lastly, it was left to the discretion of the treating interventionalist whether to strive for a mTICI 2c/3 reperfusion after mTICI 2b was achieved or end the procedure. The decision-making process for or against further retrieval attempts was not prospectively documented, but considerations might have included technical feasibility, persistent clinical deficit, procedure time, general prognosis, and remaining vascular territory at risk.

## Conclusion

Successfully improving an early mTICI 2b to mTICI 2c/3 reperfusion is possible in a substantial number of patients and might improve functional outcome. However, further retrieval attempts may increase the risk of SICH and thereby overshadow the clinical benefit of secondarily improved mTICI 2c/3 reperfusion. Yet, to support this finding, further investigation with more power is needed to account for the low number of events regarding SICH.

## Data availability statement

The raw data supporting the conclusions of this article will be made available by the authors, without undue reservation.

## Ethics statement

The studies involving human participants were reviewed and approved by Ethics Committee LMU Munich, approval number 689-15. Written informed consent for participation was not required for this study in accordance with the national legislation and the institutional requirements.

## Author contributions

PS and NV: study conception and design, data analysis and interpretation, and drafting the article. RM: critical revision of the article. MD-C and AA: data collection. GT: critical revision of the article. JF: critical revision of the article and data analysis and interpretation. FF: conception or design of the work, data analysis and interpretation, and critical revision of the article. All authors contributed to the article and approved the submitted version.

## Conflict of interest

Author GT reports personal fees from Acandis, grants and personal fees from Bayer, personal fees from Bristol Myers Squibb/Pfizer, personal fees from Boehringer Ingel-heim, personal fees from Daiichi Sankyo, personal fees from Portola, and personal fees from Stryker outside the submitted work. Author JF reports grants and personal fees from Acandis, grants and personal fees from Cerenovus, grants and personal fees from Medtronic, grants and personal fees from Microvention, personal fees from Penumbra, and personal fees from Phenox outside the submitted work, and chief executive officer of Eppdata. Author FF reports personal fees from Eppdata GmbH outside the sub-mitted work. The remaining authors declare that the research was conducted in the absence of any commercial or financial relationships that could be construed as a potential conflict of interest.

## Publisher's note

All claims expressed in this article are solely those of the authors and do not necessarily represent those of their affiliated organizations, or those of the publisher, the editors and the reviewers. Any product that may be evaluated in this article, or claim that may be made by its manufacturer, is not guaranteed or endorsed by the publisher.
